# Comparison of Electroacupuncture and Moxibustion for Relieving Visceral Hypersensitivity in Rats with Constipation-Predominant Irritable Bowel Syndrome

**DOI:** 10.1155/2016/9410505

**Published:** 2016-09-22

**Authors:** Ji-Meng Zhao, Liu Chen, Ci-Li Zhou, Yin Shi, Yu-Wei Li, Hai-Xia Shang, Lu-Yi Wu, Chun-Hui Bao, Chuan-Zi Dou, Huan-Gan Wu

**Affiliations:** ^1^Shanghai University of Traditional Chinese Medicine, Shanghai 201203, China; ^2^Shanghai Institute of Acupuncture-Moxibustion and Meridian, Shanghai 200030, China

## Abstract

*Aim*. To compare whether there is different effect between electroacupuncture (EA) and moxibustion (Mox) on visceral hypersensitivity (their analgesic effects) in constipation-predominant irritable bowel syndrome (C-IBS).* Methods*. EA at 1 mA and 3 mA and Mox at 43°C and 46°C were applied to the Shangjuxu (ST37, bilateral) acupoint in rats with C-IBS and normal rats. An abdominal withdrawal reflex (AWR) score was used to assess visceral hypersensitivity. Toluidine blue staining was used to assess mast cell (MC) activity in colon of rats. Immunochemistry was used to measure 5-HT and 5-HT4 receptor expression in the colon.* Results*. AWR scores in all EA (1 mA and 3 mA) and Mox (43°C and 46°C) treatment groups after colorectal distention (CRD) stimulation pressure of 20, 40, 60, and 80 mmHg were significantly lower than those of the model (MC) group (*P* all < 0.01). The MC counts and degranulation rates in the colon of all EA and Mox treatment groups and the MC group were significantly higher than those of the NC group (*P* all < 0.01). MC degranulation rates in the colon of all EA and Mox treatment groups were lower than those of the MC group (*P* all < 0.05). 5-HT expression in colon of all EA and Mox treatment groups was significantly lower than that of the MC group (*P* all < 0.01), and 5-HT4R expression in colon of both EA groups was significantly higher than that of the MC group (*P* both < 0.01).* Conclusion*. EA and Mox treatments may both ameliorate visceral hypersensitivity at different degree in rats with C-IBS, and EA treatment was better than Mox.

## 1. Introduction

Irritable bowel syndrome (IBS) is a common functional gastrointestinal disorder featuring abdominal discomfort or pain associated with abnormal bowel movements. IBS with constipation as a chief feature accounts for 44% of IBS cases [1]. The pathology of C-IBS is complicated but current data suggest it to be mainly caused by altered gut motility and visceral hypersensitivity [2–4]. In the pathogenesis and clinical study of C-IBS, 5-HT, 5-HT4R, and MC become the focus of research in the modern medical field. 5-HT is an important neurotransmitter and paracrine signaling molecule in the gastrointestinal tract which may directly or indirectly regulate reflective gastrointestinal motor and secretory functions and modulate intestinal pain perception under certain conditions [5, 6]. 5-HT4R is an important 5-HT receptor involved in regulating intestinal function. Activation of 5-HT4R may influence intestinal sensitivity and motility by stimulating nerve endings to release acetylcholine, or by directly targeting smooth muscle functions [7]. MCs are immune cells present in the intestinal tract that upon activation leave preformed granules to interact with local intestinal nerve fibers and mediate allergic responses through peripheral nerves that contribute to visceral hypersensitivity in IBS patients [8, 9]. Modern studies suggest that, as a type of mechanical stimulation, acupuncture works by piercing metal needles into different acupoints by certain depths and stimulating manipulations such as lifting, thrusting, and twirling way. Moxibustion is a thermal stimulation in which burning moxa produces thermal stimulation to the human body. Many studies indicate that electroacupuncture (EA) and moxibustion (Mox) can regulate C-IBS. Both techniques are reported to activate receptors or other bioactive substances at acupoints and produce nerve stimulation which is transmitted through afferent nerve fibers to the CNS and then to target organs [10–13].

In this study, we aimed to analyze whether there is different effect between EA with different current intensities and Mox with different temperatures on visceral hypersensitivity (their analgesic effects) in C-IBS. We used a C-IBS rat model to examine whether electroacupuncture at different current intensities and moxibustion at different temperatures can lead to activation of mast cells and expression changes of 5-HT and 5-HT4R in colon to relieve visceral hypersensitivity or analgesic effects.

## 2. Materials and Methods 

### 2.1. Materials

Sixty Sprague-Dawley (SD) rats (male, specific-pathogen-free, 200 ± 10 g) were supplied by Shanghai University of Traditional Chinese Medicine. Experiments were conducted strictly in accordance with the National Institute of Health Guide for the Care and Use of Laboratory Animals and the Guidelines of the International Association for the Study of Pain. All efforts were made to minimize the number of animals used and their suffering. All animal experiments in this study were performed under the guidelines approved by the Animal Ethics Committee of the Shanghai University of TCM.

### 2.2. C-IBS Model Establishment

An experimental rat model of C-IBS was established as previously study described [14]. Fifty adult male Sprague Dawley rats were given normal saline via intragastric administration (0 to 4°C, 10 mL/kg) for 14 consecutive days. Ten normal control rats received no treatment. Then, all rats were housed at a constant temperature and a humidity environment with free access to food and water. On the 28th day after the end of model development, five rats were randomly chosen from the experimental group and five rats were chosen from the normal control group to confirm that the models were successfully generated by observing their feces characteristics and abdominal withdrawal reflex (AWR) scores which was assessed by colon sensitivity to colorectal distention (CRD).

### 2.3. Treatment

Fifty C-IBS rats were randomly assigned to five groups, as follows: (1) EA 1 mA group (*n* = 10): needles (0.22 mm diameter, 13 mm length, Hwato, Suzhou Medical Appliance Factory, Ltd, Suzhou, China) were inserted 3–5 mm into the skin at the ST37 (Shangjuxu, bilateral), each acupuncture needle was connected to a HANS-100 pain relieving apparatus (Nanjing Jisheng Medical Science and Technology, Ltd., Nanjing, China) with a stimulation frequency of 2.0 Hz and a stimulation intensity of 1.0 mA. (2) EA 3 mA group (*n* = 10): the treatment was the same as EA 1 mA group but the stimulation intensity was 3 mA. (3) Mox 43°C group (*n* = 10): fine moxa made for animal experiments was ignited and placed 20 mm ± 5 mm above the acupoint. A surface thermometer (Testo 905-T2, Testo, Germany) was used to confirm the temperature (43 ± 1°C). (4) Mox 46°C group (*n* = 10): the treatment was the same as Mox 43°C group but the temperature was confirmed to be 46 ± 1°C. (5) MC group (*n* = 10): no treatment was applied but they were monitored the same as the experimental treatment groups. Ten normal rats were used as NC group.

The acupoints of bilateral ST37 were selected and EA and Mox were each applied for a total of 10 min, once daily for seven consecutive days. The ST37 acupoints of rats are located in the hind legs, about 10 mm below the fibula head at the lower outer side of the knee [15].

### 2.4. Abdominal Withdrawal Reflex (AWR) Scores before and after Treatment

After treatments, abdominal withdrawal reflex (AWR) scores were used to assess rat sensitivity to CRD using a procedure as previously study described [16]. A 2 cm balloon was made from a latex glove finger and attached to a 10 cm latex tubing that was connected to a mercury sphygmomanometer and a syringe via a three-way connector. During experiments, the balloon was coated with liquid wax and inserted slowly into the colon from the anus, about 2 cm deep, following physiological curvatures. When the balloon reached the descending colon, the rat was acclimated to the inserted balloon and air was injected into the balloon using a syringe at four different pressures: 20, 40, 60, and 80 mmHg. Each CRD lasted about 20 s. Rat AWRs were recorded blindly and expressed as the threshold of sensitivity. CRD of each intensity was repeated three times and the mean score was used as the final score. AWR scores standards are as follows: 0: no behavioral response to CRD; 1: immobility with occasional head movement; 2: mild abdominal contraction (but the abdomen did not lift from the platform); 3: a strong abdominal muscle contraction (and the abdomen lifted off the platform); 4: pelvic structures being lifted off the platform and the body arching.

### 2.5. Toluidine Blue Staining

Toluidine blue staining was used to stain MCs. First deparaffinization and hydration of 4 *μ*m paraffin-embedded sections were achieved by soaking xylenes I and II for 20 min each and anhydrous graded ethanol was applied (90, 80, and 70% for 5 min each). Then, toluidine was added to the tissue sections and stained for 20 min. Samples were washed in distilled water for 10 s and 0.5% acetic acid was added to differentiate color. Slides were observed under a light microscope until the cytoplasm turned purple-red. Samples were dehydrated with 95% ethanol for 1 min and then with anhydrous ethanol for 1 min twice. Tissues were cleared twice with xylene for 20 min each. The appropriate amount of neutral resin was added and sections were covered to be sealed. The sample slides (400x) and counted MCs were observed. Cytoplasmic granules were purple-red and nuclei were blue. Smooth and intact cytoplasmic membranes with clear nuclear staining indicated stable MCs. Broken cytoplasmic membranes with purple-red granules around cells indicated degranulated MCs. MCs were counted in three nonoverlapping random views and means were obtained for total and degranulated MCs. Degranulation rates were calculated as follows: degranulation rate (%) = degranulated MC count/total MC count × 100%.

### 2.6. Immunochemistry

The detection of 5-HT and 5-HT4R in the colonic tissue of C-IBS rats was performed by immunohistochemistry. Steps are as follows: The sections were immersed into 0.01 mol/L citrate buffer liquid (pH 6.0), microwaved at 30% power for 20 min for thermal fixing, waited for natural cooling about 20 min, and then exposed to 0.3% H_2_O_2_ for 20 min at room temperature. Following a PBS wash (3 × 3 min), 10% normal serum blocked nonspecific binding site at room temperature for 20 min. Dumping excess liquid, antibodies were added drop-wise (5-HT 1 : 100; 5-HT4R 1 : 80; Santa Cruz, CA, USA) at 37°C for 2 h and then washed 3 times with PBS for 3 min. Pig Anti-Goat IgG was added drop-wise (1 : 200) at 37°C for 30 min and then washed 3 times with PBS for 3 min. Streptavidin-HRP was added drop-wise (1 : 200) at 37°C for 30 min and then washed 3 times with PBS for 3 min. The sections were then incubated in 3,3′-diaminobenzidine (DAB) chromogenic reagent for 8–12 min and water-washed and then dyed with hematoxylin lining and water-washed. After drying, the sections were sealed with neutral gum for further observation under a light microscope.

#### 2.6.1. Image Analysis

In the fixed light intensity, we used the medical image quantitative analysis system (MIQAS) and medical image quantitative analysis software to analyze and obtain the positive area ratio of the image and optical density value (Density Optical). Positive area ratio = positive area/tissue section area, and the mean value of 3 different positive areas was measured in each slice.

### 2.7. Statistical Analysis

All statistical analyses were performed using SPSS 19.0 (SPSS Inc., Chicago, IL). AWR scores for rats are presented as interquartile ranges. Differences in means were compared by one-way ANOVA. Nonnormal data were compared using a nonparametric test. All two-sided *P* values < 0.05 were considered statistically significant.

## 3. Results 

### 3.1. AWR Score Comparisons

AWR scores of all EA and Mox treatment groups were significantly lower than those of the MC group at CRD of 20, 40, 60, and 80 mmHg (*P* all < 0.01). AWR scores of the NC group were not significantly different from the EA 1 mA and 3 mA groups at CRD pressure of 20, 40, 60, and 80 mmHg (*P* all* > *0.05), whereas AWR scores of the Mox 43°C group at 80 mmHg and the Mox 46°C group at 60 mmHg were significantly different from the NC group (*P* both < 0.05). These data suggested that EA and Mox treatments were able to decrease visceral hypersensitivity or increase the pain threshold at different degree, but EA was better than Mox treatment (Table 1).

### 3.2. Change of MC Activity in Colon

**Figure 1:**
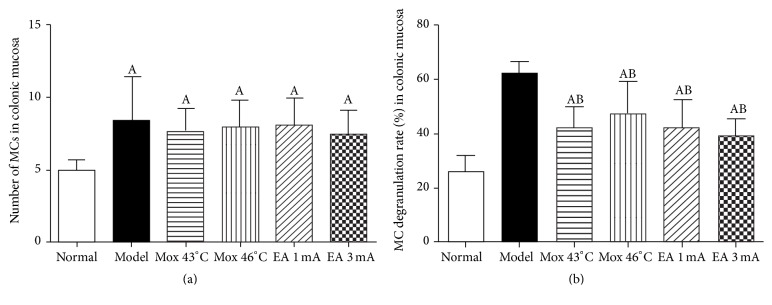
(a): Number of MCs in colonic mucosa in different group; (b): MC degranulation rate (%) in colonic mucosa in different group; ^A^ *P* < 0.01, versus normal group; ^B^ *P* < 0.05, versus model group.

**Figure 2:**
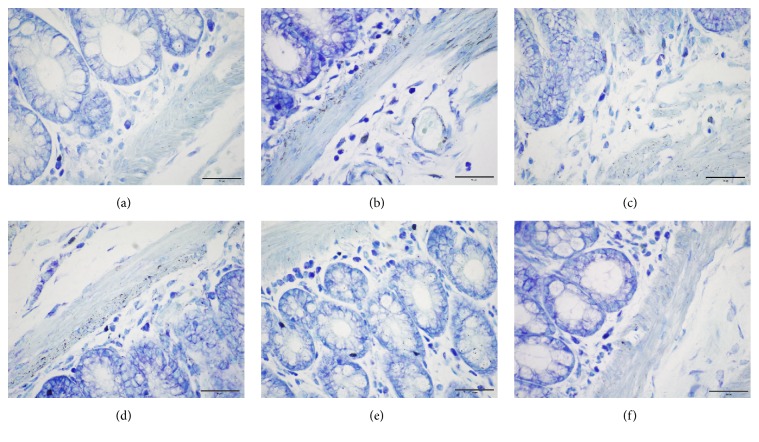
MC activity in colon in different group. (a): normal group; (b): model group; (c): Mox 43°C group; (d): Mox 46°C group; (e): EA 1 mA group; (f): EA 3 mA group.

Compared with the NC group, the MC counts in colon were significantly increased in all EA and Mox treatment groups and MC group (*P* all < 0.01). Compared with the MC group, the MC degranulation rates in colon were significantly decreased in all EA and Mox treatment groups (*P* all < 0.01). Compared with the NC group, the MC degranulation rates in colon were increased in all treatment groups (*P* all < 0.05). There was no significant difference in MC degranulation rates in all EA and Mox treatment groups among treatments (*P* all > 0.05). These data suggested that EA and Mox treatments were able to reduce the MC degranulation rates in colon (Figures 1 and 2).

### 3.3. 5-HT Expression in Colon

**Figure 3:**
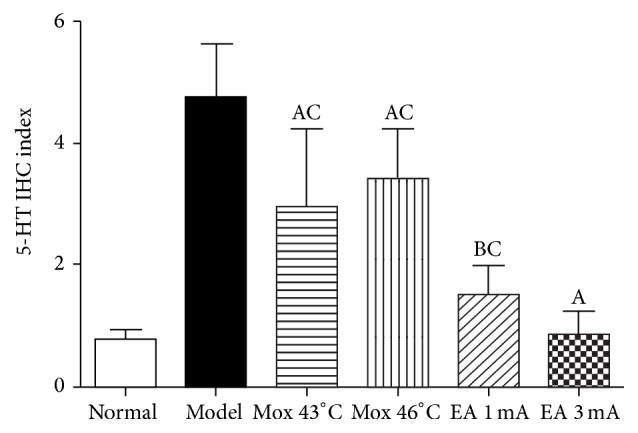
5-HT IHC index in different group. ^A^ *P* < 0.01, ^B^ *P* < 0.05, versus normal group; ^C^ *P* < 0.01, versus model group.

**Figure 4:**
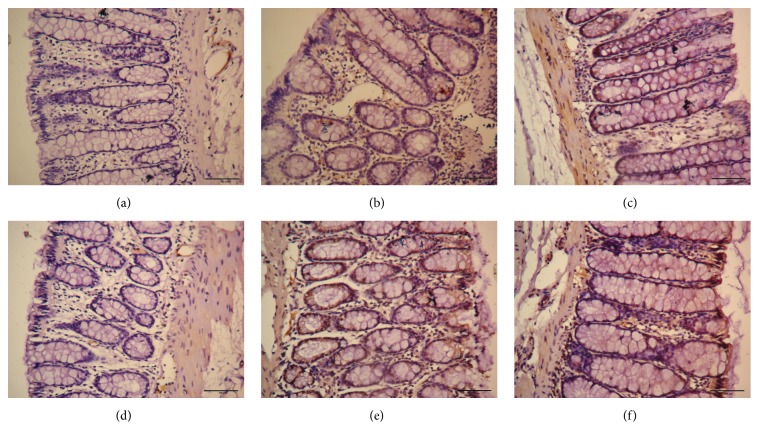
Expressions of 5-HT in colon in different group. (a): normal group; (b): model group; (c): Mox 43°C group; (d): Mox 46°C group; (e): EA 1 mA group; (f): EA 3 mA group.

Compared with MC group, 5-HT expression in colon was significantly decreased in all EA and Mox treatments group (*P* all < 0.01). Compared with NC group, 5-HT expression in colon was not different in the EA 3 mA group (*P* > 0.05) and was significantly increased in the EA 1 mA, Mox 43°C, and Mox 46°C group (*P*_EA 1 mA_ < 0.05, *P*_Mox 43°C_ < 0.01, and *P*_Mox 46°C_ < 0.01). These data suggested that EA and Mox treatments were able to reduce 5-HT expression in colon at different degree, but EA 3 mA was better than EA 1 mA, Mox 43°C, and Mox 46°C treatments (Figures 3 and 4).

### 3.4. 5-HT4R Expression in Colon

**Figure 5:**
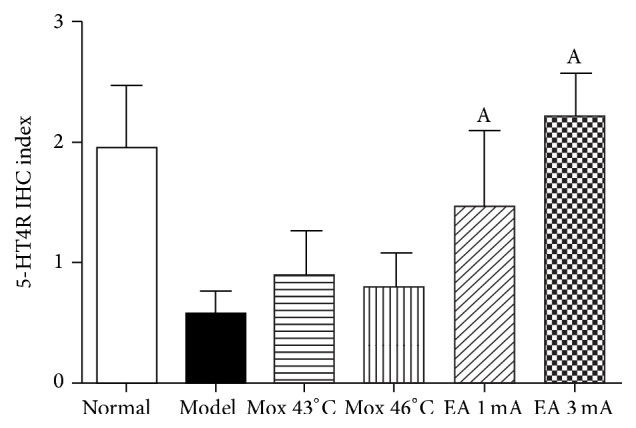
5-HT4R IHC index in different group. ^A^ *P* < 0.01, versus the model group.

**Figure 6:**
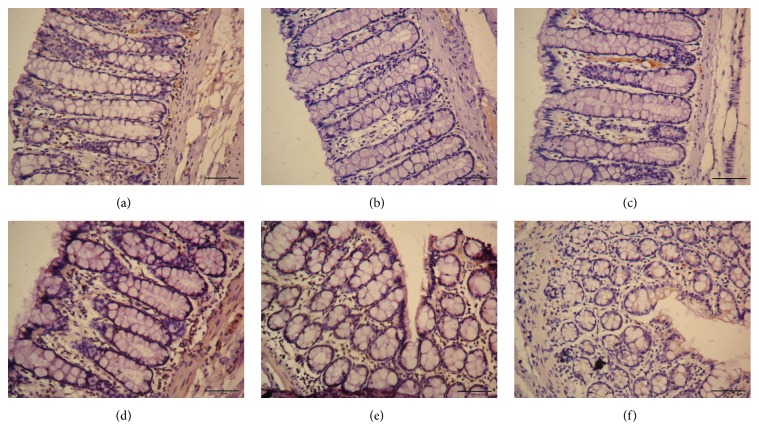
Expressions of 5-HT4R in colon in different group. (a): normal group; (b): model group; (c): Mox 43°C group; (d): Mox 46°C group; (e): EA 1 mA group; (f): EA 3 mA group.

Compared with MC group, 5-HT4R expression in colon was significantly decreased in both EA 1 mA and 3 mA groups (*P* both < 0.01), but there was no difference in both Mox 43°C and 46°C groups (*P* both > 0.05). 5-HT4R expression in the EA 3 mA group was not different from that in the NC group (*P* > 0.05). These data suggested that EA 1 mA and 3 mA treatments were able to increase 5-HT4R expression in colon, and EA 3 mA was better than EA 1 mA treatment (Figures 5 and 6).

## 4. Discussion

In recent years, with the increase of C-IBS population, the research on their pathogenesis has attracted more and more attention. Visceral hypersensitivity and abnormal intestinal motility in the brain-gut axis play a pivotal role in the pathophysiology of IBS.

As an important regulatory monoamine neurotransmitter in the brain-gut axis, 5-HT is widely present in the CNS and gastrointestinal tract where 95% of 5-HT is stored in enterochromaffin (EC) cells of the intestinal mucosa. Various stimuli such as elevated pressure in the intestinal lumen, allergic reactions, and acetylcholine can stimulate ECs to release 5-HT to signal the nervous and endocrine systems to produce visceral hypersensitivity, pain, and stool changes [17–19]. Studies suggest that, in IBS patients, an abnormal increase of 5-HT in the colon mucosa and serum is associated with visceral hypersensitivity [20–22]. We observed that 5-HT expression in MC group was significantly higher than in NC group. AWR scores of MC group were also higher than NC group, and these data suggest that overexpression of 5-HT in the colon is related to visceral hypersensitivity. After treating C-IBS rats with EA at 1 mA and 3 mA or Mox at 43°C and 46°C, colonic 5-HT expression decreased significantly, and AWR scores were lowered compared with MC group. In particular, the 3 mA current was optimal for decreasing both 5-HT expression and AWR scores, which approached that of NC group.

5-HT4R is an important 5-HT receptor that regulates gastrointestinal function [23] and 5-HT acts through 5-HT4R at the myenteric plexus to indirectly modulate gastrointestinal motility. When activated, 5-HT4R may mediate acetylcholine (Ach) or substance P (SP) motor neurons to release neurotransmitters (SP, CGRP, VIP, and NO) that are critical for regulating gastrointestinal motility and visceral hypersensitivity [24–27]. In addition, 5-HT4R is also involved in afferent gastrointestinal sensations and 5-HT may regulate visceral pain by modulating signal transduction, activating downstream inhibitory pathways to act on presynaptic dorsal horn neurons [28]. In our experiment, 5-HT4R expression in colon was significantly lower in MC group than in NC group suggesting that abnormal expression of 5-HT4R is involved in gastrointestinal motility disorders (decreased or attenuated) and visceral hypersensitivity. After treating C-IBS rats with EA at either 1 mA or 3 mA, 5-HT4R expression increased significantly and AWR scores decreased significantly. No significant difference was observed in colonic 5-HT4R expression when C-IBS rats were treated with Mox at either 43°C or 46°C. AWR scores of the Mox 43°C group at 80 mmHg and the Mox 46°C group at 60 mmHg were not significantly changed, indicating that EA was better than Mox for promoting intestinal motility and attenuating visceral hypersensitivity.

MCs are present in human and animal connective and lymphatic tissue and vessels, skin and capillaries, respiratory tract submucosa, and digestive tract [29–31]. MCs in the gastrointestinal tract can be activated by allergens, neuropeptides, and mechanical pressure. MCs, upon activation, can release these chemicals (including histamine, 5-HT, proteoglycans, platelet activating factor, cytokines, leukotrienes, and prostaglandins), which contribute to important neurological functions such as intestinal tract smooth muscle contraction and gastrocolic reflexes, but may also induce visceral sensitivity of pain [32–37]. We observed that MCs counts in colonic tissue were higher than all EA and Mox treatment groups and MC group compared with NC group, suggesting that the colonic tissue of C-IBS rats has significantly more MCs during visceral hypersensitivity. MC degranulation rate in colon was higher in all EA and Mox treatment groups than in NC group, but it was significantly reduced in all treatment groups compared with MC group; this result is consistent with a previous report from our group [15]. Thus, Ea and Mox treatments can improve visceral hypersensitivity in C-IBS rats by regulating MC activity.

In conclusion, we observed that EA and Mox treatments may both relieve visceral hypersensitivity at different degree in rats with C-IBS and produce analgesia effect, but EA treatment was better than Mox treatment. The mechanism of these effects is likely to regulate the MC activity and the expression of 5-HT in colon of rats with C-IBS. In addition, the better effect of EA treatment on relieving visceral hypersensitivity in rats with C-IBS may be associated with regulation of 5-HT4R expression in colon.

## Figures and Tables

**Table 1:** AWR scores of rats in each group after stimulation [M (P25, P75)].

Group	*n*	20 mmHg	40 mmHg	60 mmHg	80 mmHg
Normal	10	0.00 (0.00-0.00)^B^	1.00 (0.00–1.00)^B^	1.00 (1.00–1.25)^B^	2.00 (1.75–2.00)^B^
Model	10	0.75 (1.00-1.00)^D^	2.00 (2.00–3.00)^D^	3.00 (2.75–3.00)^D^	4.00 (3.00–4.00)^D^
Mox 43°C	10	0.00 (0.00–1.00)^A^	1.00 (0.00–1.00)^B^	1.50 (1.00–2.00)^B^	2.00 (2.00–3.00)^B^
Mox 46°C	10	0.00 (0.00–1.00)^A^	1.00 (1.00-1.00)^B^	1.50 (1.00–2.00)^B^	2.00 (2.00–2.25)^B^
EA 1 mA	10	0.00 (0.00–0.25)^B^	1.00 (0.00–1.00)^B^	2.00 (1.00–2.00)^B^	2.00 (2.00–3.00)^B^
EA 3 mA	10	0.00 (0.00–1.00)^A^	1.00 (0.75–1.00)^B^	2.00 (1.00–2.00)^B^	2.00 (2.00–3.00)^B^

^A^ *P* < 0.05, ^B^ *P* < 0.01, versus the normal group; ^D^ *P* < 0.01, versus model group.
